# DeepLabCut-based daily behavioural and posture analysis in a cricket

**DOI:** 10.1242/bio.060237

**Published:** 2024-04-23

**Authors:** Shota Hayakawa, Kosuke Kataoka, Masanobu Yamamoto, Toru Asahi, Takeshi Suzuki

**Affiliations:** ^1^ Department of Advanced Science and Engineering, Graduate School of Advanced Science and Engineering, Waseda University, Tokyo 162-8480, Japan; ^2^ Comprehensive Research Organization, Waseda University, Tokyo 162-8480, Japan; ^3^ Division of Sciences for Biological System, Graduate School of Bio-Applications and Systems Engineering, Tokyo University of Agriculture and Technology, Tokyo 184-8588, Japan; ^4^ Research Organization for Nano & Life Innovation, Waseda University, Tokyo 162-0041, Japan

**Keywords:** Cricket, Circadian rhythm, Sleep-like state, Machine learning, Posture analysis

## Abstract

Circadian rhythms are indispensable intrinsic programs that regulate the daily rhythmicity of physiological processes, such as feeding and sleep. The cricket has been employed as a model organism for understanding the neural mechanisms underlying circadian rhythms in insects. However, previous studies measuring rhythm-controlled behaviours only analysed locomotive activity using seesaw-type and infrared sensor-based actometers. Meanwhile, advances in deep learning techniques have made it possible to analyse animal behaviour and posture using software that is devoid of human bias and does not require physical tagging of individual animals. Here, we present a system that can simultaneously quantify multiple behaviours in individual crickets – such as locomotor activity, feeding, and sleep-like states – in the long-term, using DeepLabCut, a supervised machine learning-based software for body keypoints labelling. Our system successfully labelled the six body parts of a single cricket with a high level of confidence and produced reliable data showing the diurnal rhythms of multiple behaviours. Our system also enabled the estimation of sleep-like states by focusing on posture, instead of immobility time, which is a conventional parameter. We anticipate that this system will provide an opportunity for simultaneous and automatic prediction of cricket behaviour and posture, facilitating the study of circadian rhythms.

## Teaser

**Summary:** The cricket has been employed to study the neural basis of circadian rhythms. This study presents a label-free system for measuring diurnal rhythms of behaviours based on supervised machine learning.

## INTRODUCTION

Circadian rhythms are endogenous oscillations observed in most animals. Circadian rhythms have a free-running period of approximately 24 h, entrained by daily environmental cues, such as light and temperature. In insects, circadian rhythms control the daily rhythmicity of physiological and developmental processes, such as hatching, pupation, and moulting, as well as behaviour (e.g. locomotion, feeding, sleep, mating, and oviposition) (Saunders, 2002).

Recent advances in computer vision and deep learning have led to several open-source programs and commercial systems that accurately, rapidly, and robustly measure animal behaviour. Videography is the most common and widely used method for measuring animal behaviour because it is non-invasive and provides high-resolution behavioural observations. Although the extraction of animal behaviour from videos poses a computational challenge, recent developments in computer vision have offered numerous algorithms for processing image data. In particular, supervised machine learning methods that automatically label and track ‘keypoints’ on an animal's body can identify complex behavioural changes over time. These resources not only overcome the enormous time and effort involved in manually identifying behavioural traits but also eliminate human biases and oversights during annotation. This allows us to capture multiple behaviours and behavioural action sequences in a consistent and objective manner (Anderson and Perona, 2014), offering the potential to track a variety of behaviours under circadian control.

The above-described technological advancements facilitate further research into sleep, defined by the behavioural characteristics of invertebrates. The sleep-like state (SLS) is generally defined by the following five features: (i) increased arousal threshold, (ii) reversibility, (iii) homeostasis, (iv) decreased movement, and (v) a specific sleep posture (Campbell and Tobler, 1984). The latter is accompanied by reduced muscular activity, which has been observed in nearly all animals, from mammals to arthropods (sloths: De Moura Filho et al., 1983; horses: Dallaire, 1986; bees: Kaiser, 1988; cockroaches: Tobler and Neuner-Jehle, 1992; nematodes: Schwarz et al., 2012). For fruit flies, a behavioural measurement system, called the *Drosophila* Activity Monitor System (DAMS), was previously utilised to define SLS as continuous immobility lasting for more than 5 min (Shaw et al., 2000; Hendricks et al., 2000). Continuous immobility time has been used as an evaluation metric for the SLS of insects (Ajayi et al., 2022).

Crickets have served as a useful model animal for understanding the neural mechanisms underlying circadian rhythms in insects because they have relatively large nervous systems with identifiable neurons (Cymborowski, 1973; Abe et al., 1997; Tomioka and Abdelsalam, 2004; Tomioka, 2014). Crickets are suitable for manipulation through reverse genetic methods, such as RNA interference and genome editing (Mito et al., 2011; Watanabe et al., 2012). In the context of circadian rhythms, crickets have been suggested to exhibit a unique clock oscillation mechanism in which both the *cycle* and *clock* genes are rhythmically regulated (Uryu et al., 2013). Further, considerable advances have been made in cricket genomics (e.g. *Laupala kohalensis*: Blankers et al., 2018; *Teleogryllus oceanicus*: Pascoal et al., 2020; *T. occipitalis*: Kataoka et al., 2020; *Gryllus bimaculatus*: Ylla et al., 2021; *Acheta domesticus*: Dossey et al., 2023; *Apteronemobius asahinai*: Satoh et al., 2021). These have enabled a more comprehensive study of circadian rhythms from a molecular biological perspective (Kataoka et al., 2022). An in-depth analysis of the behavioural rhythms of crickets is expected to facilitate the identification of novel roles for genes that determine circadian rhythm-controlled physiological behaviour. Previous studies measuring the daily rhythms of behaviour in crickets only adopted conventional analytical methods, such as the seesaw-type actometer (Moriyama et al., 2008) and an infrared sensor-based actometer (Yamano et al., 2001). However, these systems cannot measure activities other than locomotor activity.

In this study, we propose a method for quantifying and analysing multiple behaviours controlled by circadian rhythms, including locomotor activity, feeding, and SLS, in a cricket. To this end, we applied the open-source software DeepLabCut (Mathis et al., 2018; Nath et al., 2019; Lauer et al., 2022), which is based on the Convolutional Neural Network (CNN) of supervised machine learning, to automatically label the keypoints of cricket bodies and derive quantitative data from the coordinates of body parts. Unlike fruit flies, crickets can be accurately labelled at key points on their body parts, owing to their large body sizes. This means that the system can automatically acquire not only quantitative measurements of behavioural activities such as locomotion, feeding, and SLS, but also more complex information such as posture. In light of this advantage, we attempted to measure SLS based on posture, in addition to immobility time. Multiple behaviours were automatically detected from long-term recordings using an automated analysis package that can be easily implemented for a variety of animals. Furthermore, we propose a new method that contributes to a multifaceted definition of SLS that does not rely on restricted criteria, such as immobility time.

## RESULTS

### High-precision keypoint detection

**Fig. 1.**
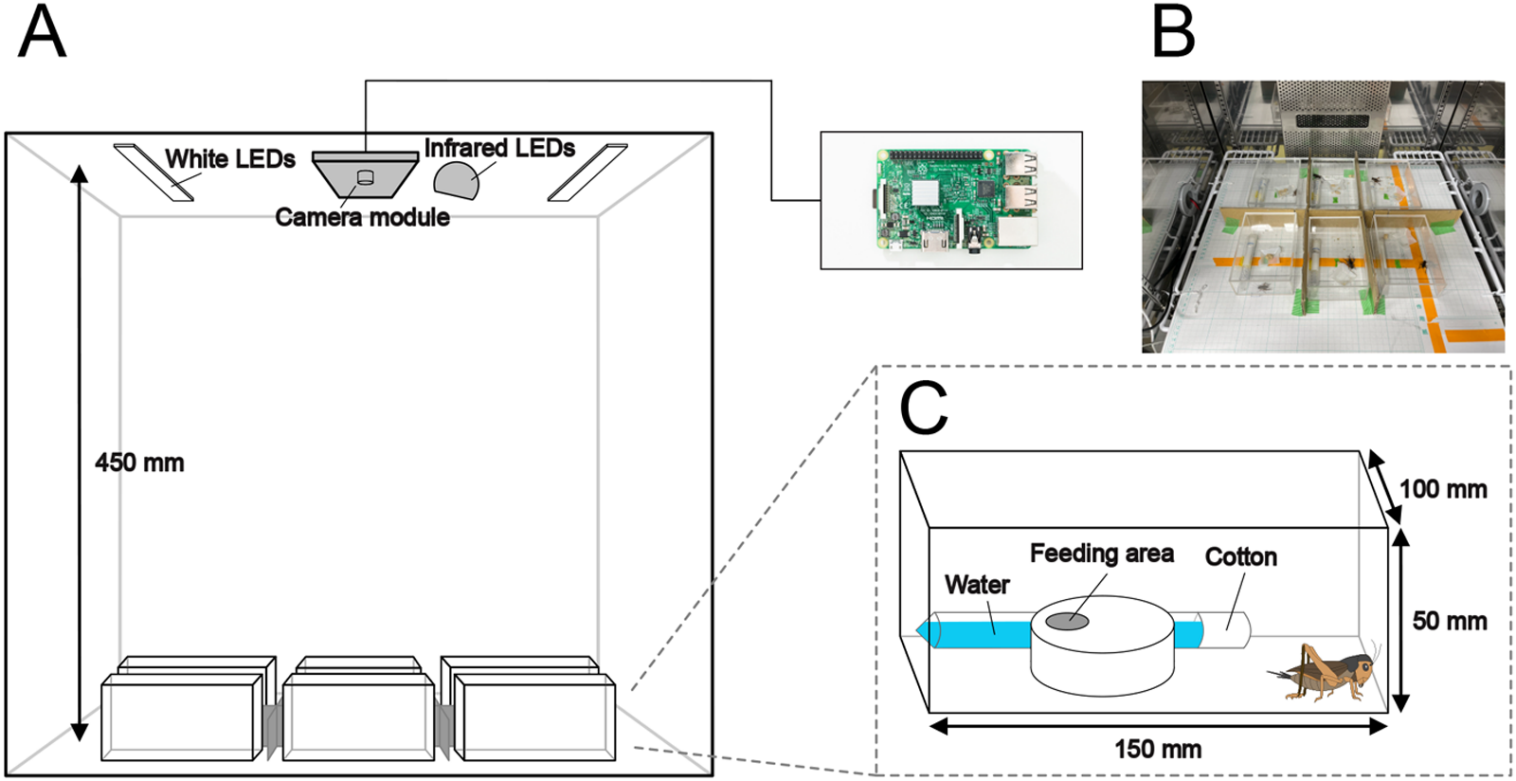
**A schematic of the cricket behaviour-recording system.** (A) An overview of the quantitative evaluation system for recording, through which we simultaneously recorded up to six crickets in one experiment by isolating individuals using acrylic cases (150×100×50 mm). The 12-h light/dark cycle (LD 12:12) was controlled by an electric time switch. The movement of crickets was recorded by a video camera, set at a height of 450 mm above the floor and connected to a Raspberry Pi 3. (B) A photograph depicting the specific recording environment. Sheets of paper with a height of 15 mm were placed between each acrylic case to make the individuals invisible to each other. (C) A schematic diagram of the acrylic case. The crickets were allowed free access to water and food. The water source is the tube-stuffed cotton, and the feeding area consists of a section of the plastic case with holes.

We set up an imaging system controlled by Raspberry Pi for the long-term quantification of a variety of behaviours in six adult crickets (Fig. 1). We imaged the crickets, each contained in an acrylic case, once per min under 12 h light 12 h dark conditions (12:12 LD) for 14 days. The resulting serial images were cropped into individual compartments and converted into videos for DeepLabCut (Movie 1). In DeepLabCut, we manually labelled the keypoints of body parts (i.e. head, thorax, abdomen, abdominal tip, left hind leg, and right hind leg) in randomly extracted frames. A ResNet50 neural network was trained using the manually labelled images (training process). The resulting model was applied to test the newly extracted frames, and the labelling predicted by this model was manually corrected (refinement process). The training and refinement processes were repeated five times. The corrected labelling generated by the refinement process was used for subsequent training. Although the losses in our models did not converge close to zero in the first three training sessions, they converged near zero after the fourth session (Fig. S1A). We used the average of the absolute Euclidean distances to compare the keypoints generated by the model with those labelled by humans. This distance was obtained by calculating the Euclidean distance between keypoints generated by the model and those labelled by humans, for every frame. We computed the training error generated during training and the test error generated during prediction using the model (Fig. S1B). The training and test errors for the first model were 3.32 px and 5.59 px, respectively (Fig. S1B). In the final model, the training and test errors were reduced to 1.61 px and 1.58 px, respectively, which are nearly equivalent (Fig. S1B,C). These results suggest that accurate body part predictions were achieved in crickets after five rounds of training and correction of model prediction.

Using the final model, we predicted six keypoints for crickets in all serial images over a 14-day period. To evaluate the labelling accuracy, the ratio of each keypoint with a likelihood greater than 0.95 was calculated for all frames (Fig. S1D). In all individuals, accurate labelling was achieved in the head, thorax, abdomen, and abdominal tip in more than 90% of frames (96.39±1.45% for head, 97.31±1.42% for thorax, 97.27±1.68% for abdomen, and 97.57±0.83% for abdominal tip). In the left hind leg and right hind leg, there tended to be fewer frames with a likelihood greater than 0.95 than in the other four points (91.59±1.70% for left hind leg, and 93.20±1.34% for right hind leg). We also checked for differences in labelling accuracy between the dark and light periods. There was a trend toward reduced labelling accuracy during the dark period for all six key points (Fig. S1E). The decrease in accuracy in the left and right hind legs was particularly remarkable (Fig. S1E). These results were likely due to the reduced resolution of images acquired under infrared light conditions.

### Locomotor activity

**Fig. 2.**
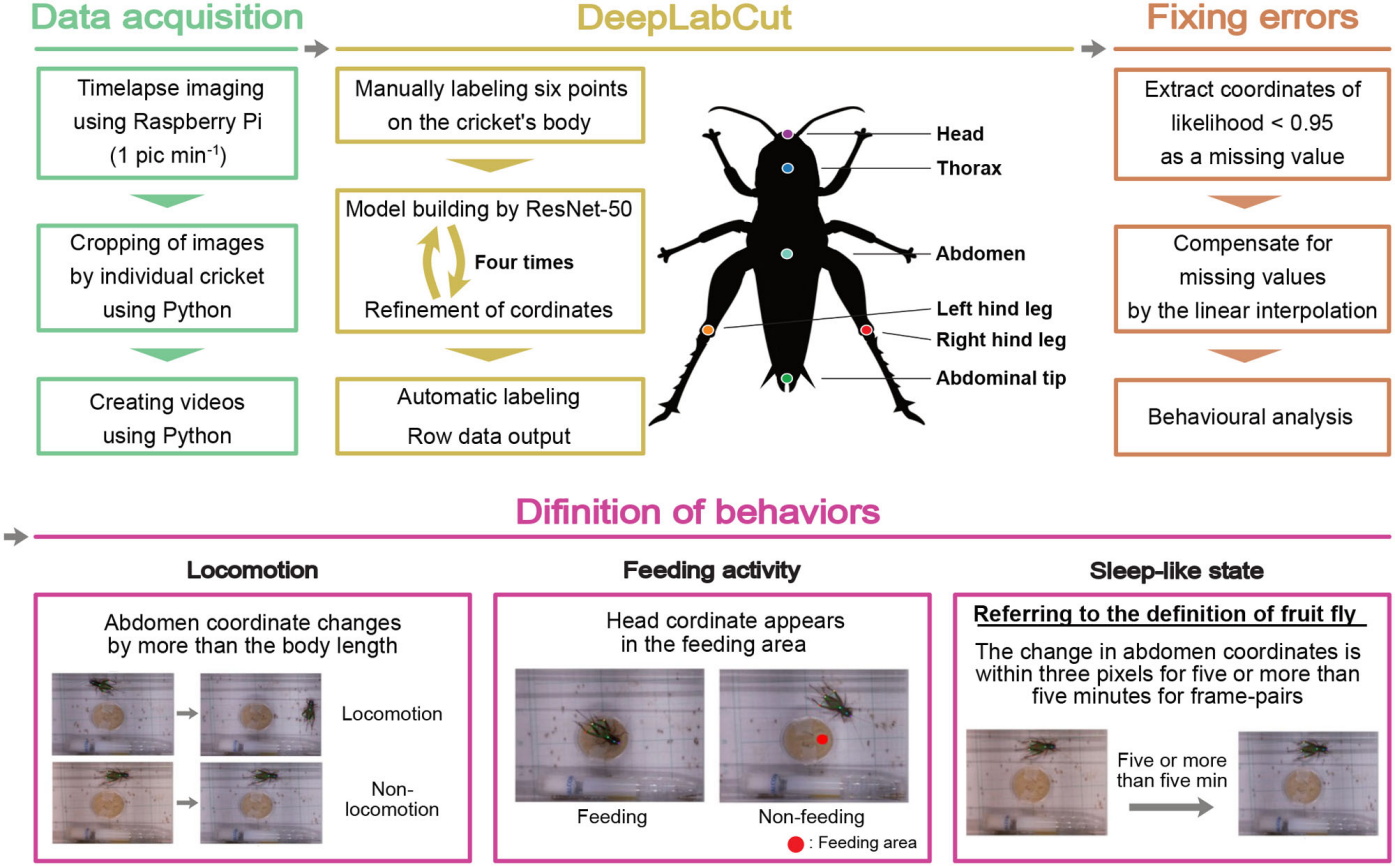
**Outline view of the system.** Scheme from image acquisition to behavioural quantification. First, image data were recorded with a camera module connected Raspberry Pi at an interval of one image per minute. Images were converted into videos, and a machine learning algorithm, ResNet-50 and DeepLabCut, was employed for training and refinement to construct a model with six automatically labelled keypoints on crickets’ head, thorax, abdomen, abdominal tip, left hind leg, and right hind leg. Subsequently, the coordinates of these labelled keypoints were extracted. After fixing errors, behaviours such as locomotion, feeding activity, and SLS were defined based on the coordinates and behaviours quantitatively analysed across all frames over a period of 14 days.

**Fig. 3.**
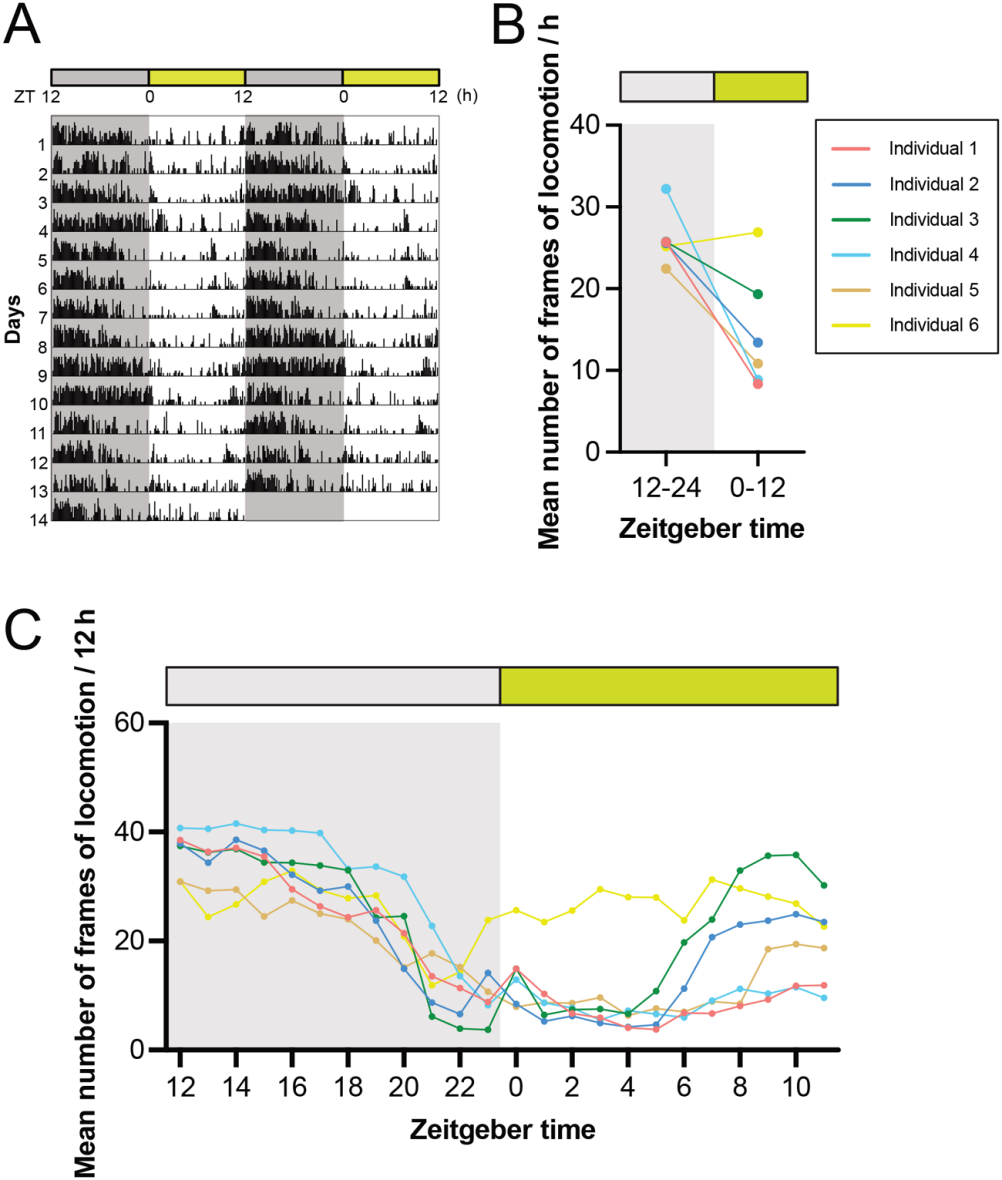
**A quantitative analysis of locomotion of crickets.** (A) The double-plotted actogram recorded under a 12-h light:dark (LD) cycle at 30°C for 14 days shows that the frequency of locomotion increases during the dark phase. (B,C) Mean number of frames of locomotion for 12 h (B) and 1 h (C) indicated that locomotion significantly increases during the dark phase (1 frame per min).

Locomotor activity was recorded for the six crickets during exposure to 12:12 LD for 14 days. The criterion for judging locomotion was defined as whether the coordinates of the abdomen moved more than a body length (from the head to the abdominal tip) within a single frame (Fig. 2). A double-plotted actogram was generated based on the data from all frames (Fig. 3A). They showed a clear nocturnal rhythm, with weak activity that started several hours before turning off the lights (Fig. 3B,C), which is consistent with previous studies in other cricket species (*Acheta domestica*: Cymborowski, 1973; *Gryllodes sigillatus*: Abe et al., 1997; *G.*: Tomioka and Abdelsalam, 2004). For most individuals, acrophases (circadian peak time) of the daily rhythms of locomotion were observed immediately after lights-off (Fig. 3C; Fig. S2A). The circadian rhythms of most animal locomotion were entrained to nearly 24 h (1440 min) by the 12:12 LD photocycle, as demonstrated by Chi-square and Lomb-Scargle periodograms (Fig. S2B). These findings indicate that the quantification of locomotor activity using DeepLabCut produces reliable results comparable to those of other available methods.

### Feeding activity

**Fig. 4.**
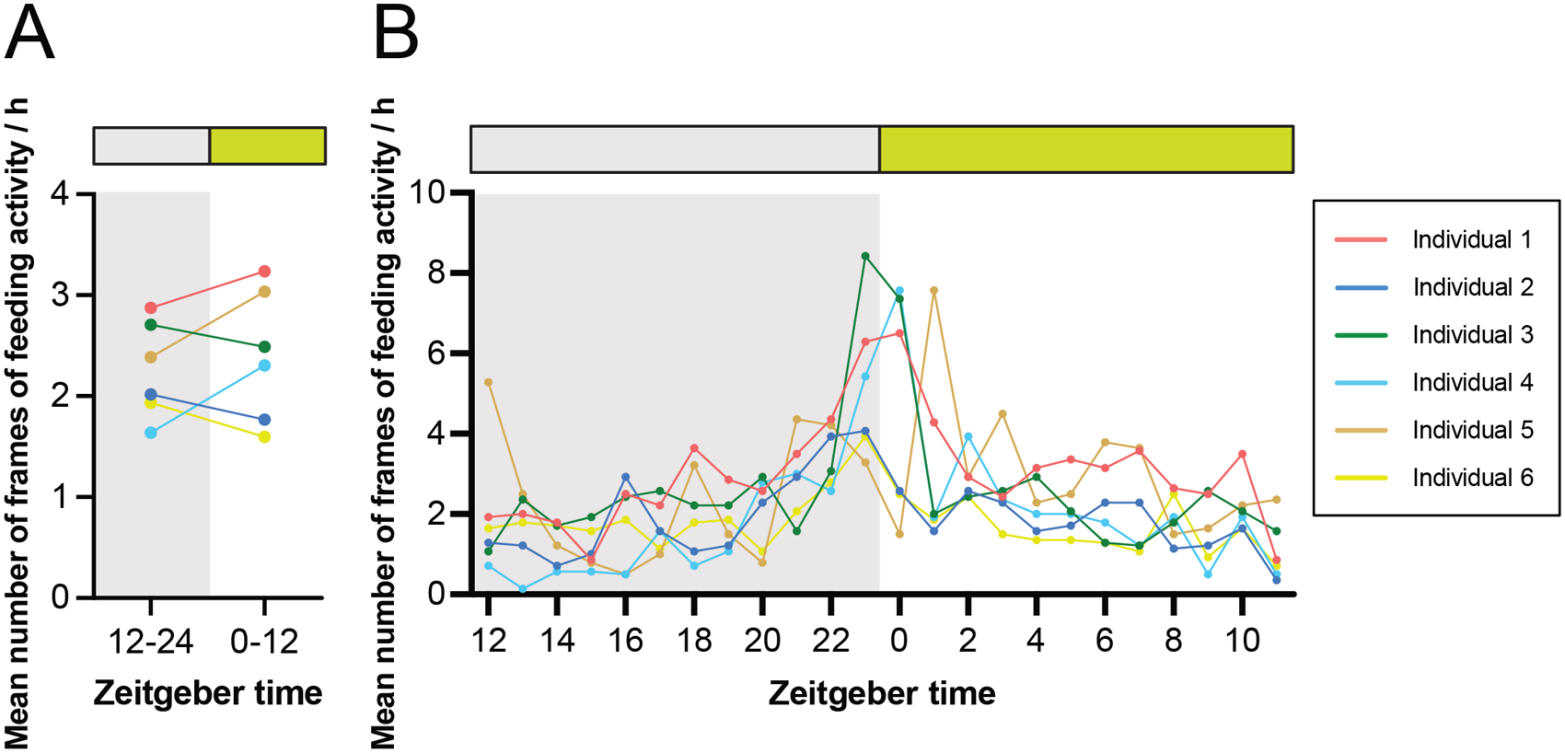
**A quantitative analysis of feeding activity of crickets.** (A,B) Mean number of frames of feeding activity for 12 h (A) and 1 h (B) suggested that peaks were evident around the transition from dark to light, though there was no significant difference in feeding activity between the dark and light phases (1 frame per min).

The diurnal rhythm of the feeding activity in crickets in a free-feeding environment was evaluated by counting the frames in which the head position overlapped with the feeding area (Fig. 2). Due to the limited number of feeding events, it was not possible to generate double-plotted actograms and periodograms. There was no significant difference in feeding activity between the dark and light phases (Fig. 4A). Nevertheless, acrophases of daily feeding activity rhythms clearly peaked around the transition from the dark to the light phase (Fig. 4B; Fig. S2A). Three out of six individuals showed an increase in feeding activity just before switching from dark to light, and their activities remained at a peak immediately after switching to light (Fig. 4B). The other three animals exhibited a peak in feeding activity either immediately before or after the switch from light to dark (Fig. 4B). Increased feeding activity during the transition between light and dark phases has also been reported in *Drosophila melanogaster* (Seay and Thummel, 2011; Ro et al., 2014; Niu et al., 2021). In *D. melanogaster*, feeding activities are concentrated close to lights-on, lights-off, or both (Seay and Thummel, 2011; Ro et al., 2014; Niu et al., 2021), possibly reflecting differences in the optimal timing of feeding activity between species. These results show that the quantitative analysis of feeding activity rhythms can be achieved by applying the prediction of body part position using DeepLabCut.

### The SLS

**Fig. 5.**
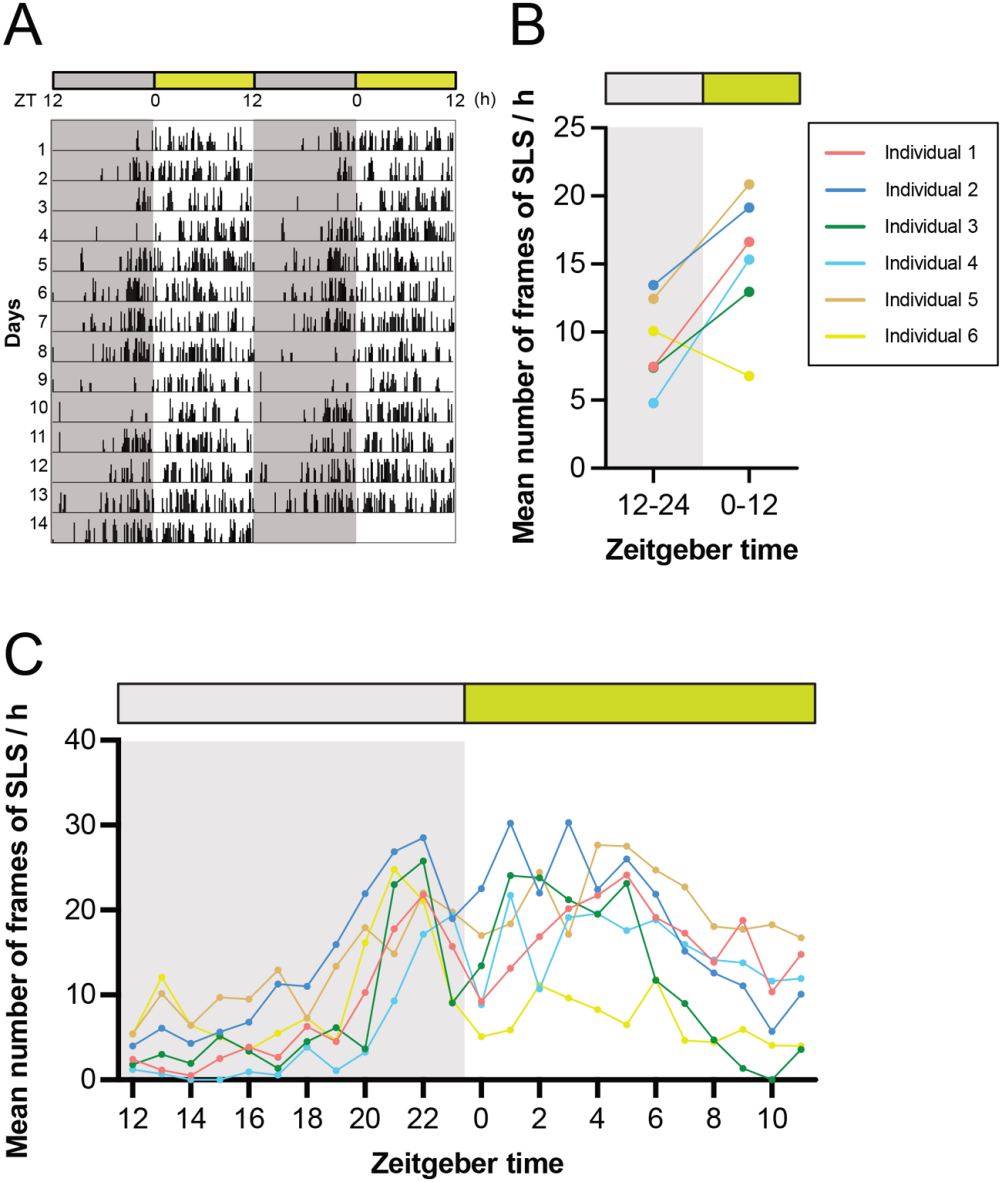
**A quantitative analysis of SLS of crickets.** (A) The double-plotted actogram recorded under a 12-h light:dark (LD) cycle at 30°C for 14 days shows that the frequency of SLS increases during the light phase. (B,C) Mean number of frames of SLS for 12 h (B) and 1 h (C) indicated that the SLS significantly increases during the light phase, with a second peak in the late dark phase (1 frame per min).

Next, diurnal rhythms in the SLS of crickets were quantified. Using the definition of the SLS in *D. melanogaster* (an immobility time of more than 5 min), we adapted this to measure continuous immobility as being more than five frame-pairs of cricket immobility (Fig. 2). In contrast to the diurnal rhythm of locomotor activity, greater immobility was observed during the light period (Fig. 5A,B). For most animals, the acrophases of the daily immobility rhythm peaked several hours after the transition from the dark to the light phase (Fig. S2A). Consistent with the locomotor activity cycles, the rhythm of the immobility was also entrained to nearly 24 h (1440 min) by the 12:12 LD photocycle (Fig. S2B). We also observed a bimodal rhythm in which a peak of the immobility was noted just before the end of the dark phase, in addition to the peak after lights-on (Fig. 5C).

**Fig. 6.**
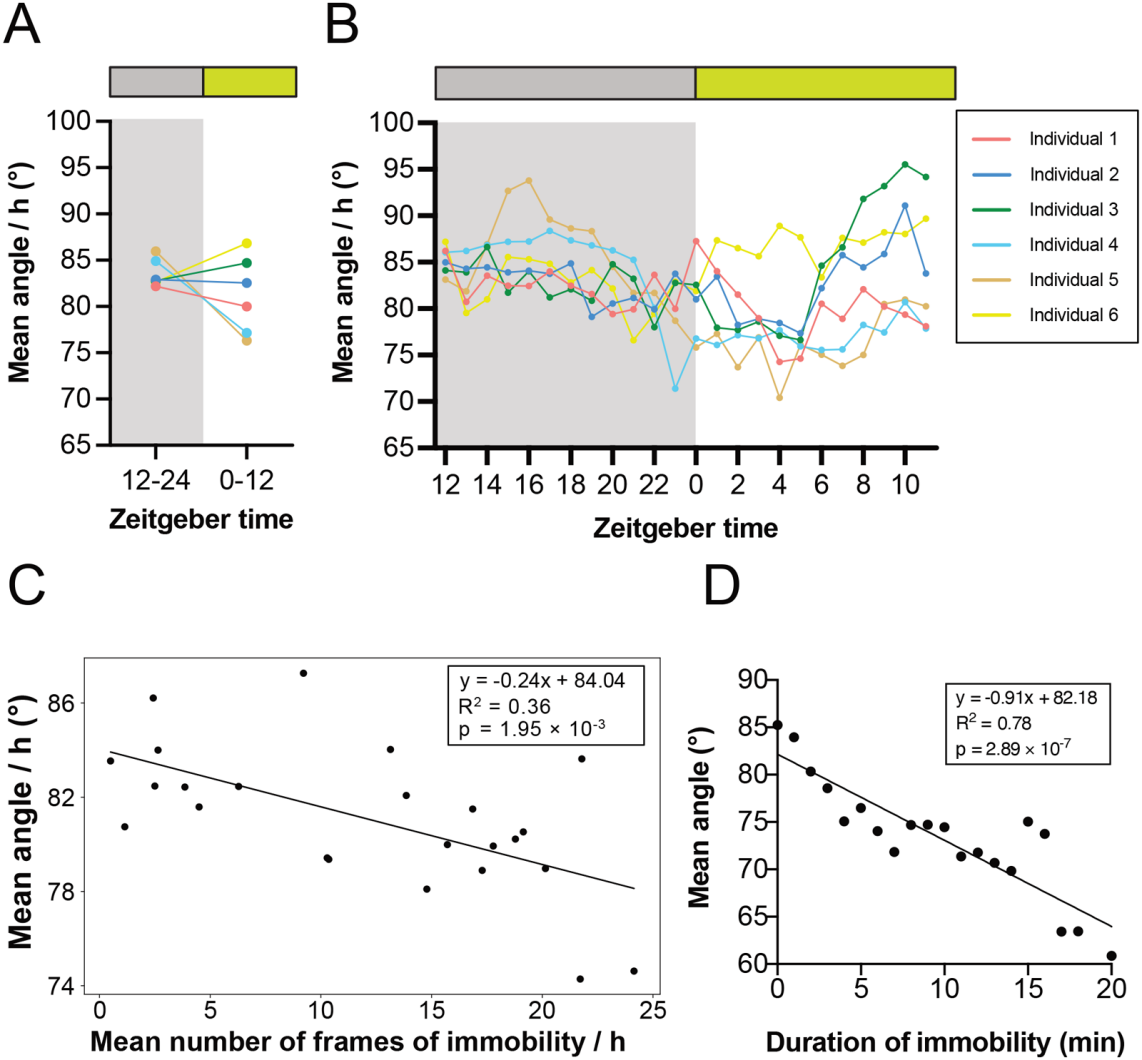
**Body posture associated with the SLS in crickets.** Body posture and leg relaxation were calculated from the angle between three points: the abdomen, left hind leg, and right hind leg. The relationship with the SLS was then examined. (A,B) The mean angle for 12 h (A) and 1 h (B) indicated that leg relaxation was significantly increased during the light period, when the frequency of the SLS was higher. (C) A scatter plot of the mean angle per hour against the mean SLS frequency corresponding to same hour was calculated with a fitted linear regression line. The equation of the line, with R squared as the coefficient of determination and *P*-value, indicates a negative correlation between hind legs angle and the number of continuous immobility frames. (D) Mean hind legs angle was calculated for each immobile time from 0 to 20 min with a fitted linear regression line. The equation of the line, with R squared as the coefficient of determination and *P*-value, indicates a strong negative correlation.

The behaviour-tracking system utilising DeepLabCut is particularly suitable for strictly defining the animal SLS based on a well-characterised sleeping posture (Campbell and Tobler, 1984). During the active phase, crickets support their bodies using the skeletal muscles, while during the SLS, their hind legs are relaxed to lying position along with the antennae drooping (Fig. S3A). This characteristic position may be associated with the SLS in crickets (Nishio, 2009). To quantify the diurnal rhythm of the SLS based on hind leg relaxation, we analysed the angle formed by three points from the coordinates of the abdomen, left hind legs, and right hind legs, which were predicted using DeepLabCut for each frame (Fig. S3A). Four out of the six individuals showed a smaller hind leg angle in the light phase than in the dark phase, suggesting leg relaxation during the former phase (Fig. 6A,B). To investigate the correlation between hind leg angle and the number of continuous immobility frames, we performed the following two analyses. First, we calculated the average angle of the hind legs and the average frames of SLS for the same 1-h zeitgeber time over 14 days, investigating their correlation. The results revealed a negative correlation between the hind leg angle and SLS in all individuals (Fig. 6C; Fig. S3B). Second, the average angle of the hind leg was calculated for immobility durations starting from 0 min and increasing in 1-min increments, up to 20 min per individual. The results show that the longer the continuous immobility, the smaller the hind leg angle (Fig. 6D; Fig. S3C), but the individual differences were larger than in the first analysis. Collectively, these data suggest that hind leg angle tends to be negatively correlated with an individual's immobility and that postural assessment based on hind leg angle may be useful for estimating SLS more precisely.

## DISCUSSION

In this study, we developed a system using the DeepLabCut to automatically analyse the diurnal rhythms of behaviours and postures of crickets, which are a well-established model organism for studies into the circadian rhythm and behaviour of hemimetabolous insects. Our system enabled us to simultaneously measure the rhythms of locomotive activity, in addition to feeding behaviour and SLS rhythms in a single experimental run. DeepLabCut has provided analysis of the diurnal rhythm of SLS by postural estimation based on hind legs, as well as by the conventionally adopted criterion of at least 5 min of continuous immobility time in *D. melanogaster* (Shaw et al., 2000; Hendricks et al., 2000). Our system is expected to save researchers a great deal of time, generate reproducible results by eliminating inter-researcher variability, and enable experiments that are not possible with other methods, such as the seesaw-type actometer (Moriyama et al., 2008) and the infrared sensor-based actometer (Yamano et al., 2001).

Our study successfully leveraged DeepLabCut to automatically label cricket body parts with high accuracy, but low-confidence labels (likelihood <0.95) were included at approximately 5% or less during the light phase and at about 5-15% during the dark phase. Although the arena for behavioural experiments used in this study was designed to be user-friendly and easy to set up, low-confidence labels were believed to be generated by the reflection of light from LEDs on the acrylic case and by the shadows cast by objects in the acrylic case. In this study, low-confidence labels with likelihood <0.95 were treated as missing values and supplemented with the mean values of the pre- and post-frames. The environment setting and number of training sessions of the model should be determined to ensure the spatial resolution required to detect the behaviour and posture of interest to researchers. To improve the accuracy of labels for frames collected in the dark, it would be necessary to increase the number of IR lights, improve camera functionality, and adjust the height of the water and feeding areas to reduce shadows.

In this study, we applied the SLS criterion used in *D. melanogaster*, ‘continuous immobility for at least 5 min’, to crickets as ‘continuous immobility over five frames’ and were able to quantify the diurnal SLS rhythms. Moreover, we were able to quantify hind leg relaxation based on the positional coordinates predicted by DeepLabCut, allowing us to analyse the SLS in a different manner than in previous studies. SLS was observed at high levels during the light phase but also showed an additional transient peak in the late dark phase, consistent with previous studies in *Drosophila* (Dubowy and Sehgal, 2017). Analysis of the SLS rhythm focusing on hind leg relaxation showed that the average angle of the hind legs was smaller in many individuals during the light period. Notably, there was a decrease in the average angle of the hind legs in the late dark phase but only in approximately half of the individuals used in this experiment, suggesting that crickets may have had short bouts of siesta sleep during the dark phase (active phase). Thus, it is possible that the SLS observed during the late dark phase and those observed during the light phase (inactive phase) may have different physiological significance. Future investigation would require careful observation of individuals at a fine temporal resolution, focusing on movements such as posture, tactile sensation, and digestion.

There tended to be a negative correlation between hind leg angle and immobility duration, suggesting that the hind leg angle can be an indicator for detecting SLS. However, there was variation in the relationship between the angle of the hind legs and the length of continuous immobility frames among individuals, suggesting that more multifaceted analysis is needed. In addition, the thresholds for homeostasis, arousal, and immobility duration, which are characteristics of the SLS, may differ among species (Allada et al., 2017; van Alphen et al., 2013). Furthermore, it is helpful to perform electrical brain measurements to verify whether the observed SLS is true sleep. In the future, homeostatic assessment and electrophysiological approaches will be required to advance sleep research in crickets.

In summary, we report the DeepLabCut-based accurate detection of multiple behaviours and postures over 2 weeks in multiple crickets. Our system can be easily applied to various animal species other than crickets and allows for the evolutionary study of many physiological processes, including sleep. By combining high-fidelity behavioural tracking with genomic and transcriptomic data, it is possible to explore and understand the molecular basis of behavioural variations and phenotypic plasticity. Furthermore, enhancing temporal resolution enables detection of insect behaviours, such as grooming and courtship, which supports analysis of interrelation of multiple types of behaviours. This analysis also opens the possibility of detecting abnormal behaviours such as infection and sleep disorder, as well as predicting in advance the individuals who exhibit these abnormal behaviours.

## MATERIALS AND METHODS

### Animals

All experiments were performed with crickets (*Teleogryllus occipitalis*) that were collected in Amami Oshima Island, Kagoshima, Japan, and cultured in a laboratory, maintained under standard environmental conditions of 12 h light and 12 h darkness (LD 12:12, light: 0600-1800 h; Japan Standard Time) at a constant temperature of 30°C. Adult virgin female crickets were reared individually from the last instar stage and were used for recording 0 or 1 day after emergence. The animals were provided with cricket food (Tsukiyono Farm, Gunma, Japan) and water *ad libitum*. In the recordings, six individuals were placed individually in their cases, and all six were simultaneously recorded. The reason for using virgin females in this study was to prevent the chirping sounds produced by adult males from influencing others.

### Recording system

The recording system used in this study is shown in Fig. 1. The crickets were individually placed in transparent acrylic cases (150×100×50 mm). To render the individuals invisible to each other, sheets of paper with a height of 15 mm were placed between each acrylic case. Imaging was conducted using an infrared camera module (Raspberry Pi NoIR; 3280×2464 pixels) connected to a Raspberry Pi 3 model B (Raspberry Pi Foundation, Cambridge, UK), which captured RGB images of the crickets at 1-min intervals. The camera was positioned 450 mm above the bottom of the acrylic case. The experimental system was installed inside an incubator with an air temperature maintained at 30°C, and humidity was kept at 45±5%. Illumination was performed using white LEDs (Timely, Tokyo, Japan) and infrared LEDs (Broadwatch, Tokyo, Japan). The white LEDs were connected to a time switch, ensuring a 12-h light and a 12-h dark period (LD 12:12; light period: 6:00 to 18:00, Japan Standard Time; 0 to 12, Zeitgeber Time). Light intensity at the position of the animals was measured using a spectroradiometer (MS-720; Eko Instruments, Shibuya, Tokyo, Japan) and was approximately 150 lux. After entraining the crickets to a cycle of LD 12:12 light cycle for 3 days (not recorded), 14-day time-lapse imaging was conducted. During this period, food and water replenishment and replacement were carried out three times, at intervals of 3 and 4 days, respectively.

### Data acquisition

All images obtained were cropped such that each acrylic case for each individual fit (Fig. 2). A series of images were created as a single movie and fed into DeepLabCut. The lens distortion, which depends on the distance and angle between the camera and the subject, was evaluated based on 10 mm×10 mm grid squares placed under the acrylic cases. The maximum difference in length owing to lens distortion at the centre point and the four corners of the image was less than one pixel. This corresponded to 0.68% of the body length of the crickets used in this experiment. Furthermore, this was smaller than the average offset from automatic labelling via machine learning, as described later. Therefore, it was concluded that lens distortion in this recording environment did not affect behaviour quantification, and correction for lens distortion was not performed.

### DeepLabCut training and evaluation

We used DeepLabCut for the MacOS M1 chip (i.e. DEEPLABCUT_M1) to quantify cricket locomotion, feeding activity, immobility time, and posture. The aim was to construct a model that could automatically estimate six points on the body of a cricket: the head, thorax, abdomen, abdominal tip, left hind leg, and right hind leg (Fig. 2). The abdomen point was defined as a midpoint between the contact points of the femurs of the left and right hind legs and the body from a top-down perspective. To generate training datasets for the first training, 120 frames were randomly sampled from each video using k-means clustering in DeepLabCut. Six points on the body of the cricket were manually labelled. In the first training step, a ResNet-50-based neural network with 50,000 iterations was used. Because the model performance was initially suboptimal, we refined the model after the first training. During refinement, the keypoints labelled in 120 randomly extracted frames were manually corrected. These training and refinement processes were repeated four times, culminating in five training sessions to construct the final model. The number of iterations was set to 50,000 from the first to third sessions, 200,000 from the fourth session, and 500,000 from the fifth session.

To evaluate the training of the model, the relationship between the iteration number and the loss value was calculated. Next, the mean deviation (px) of the coordinates of the six keypoints between the manually labelled teacher data and automatic labelling by the model was calculated for each training set as a training error. Simultaneously, the mean deviation (px) of the coordinates of the six keypoints between the manually labelled data without the teacher data and automatic labelling was calculated for each training set as a test error. Additionally, the proportion of data with a likelihood value of 0.95 or higher and less than 0.95 as missing values, representing the estimated probability for each keypoint, was calculated to assess the labelling accuracy across all data. When the keypoints of a cricket were obscured by another object, the model attempted to approximate its position. The likelihood value of such frames is typically low. In this study, coordinates with a likelihood value of less than 0.95 were removed. The missing values were imputed by mean imputation using a Python script (Fig. 2).

### Behaviour analysis

Locomotor activity, feeding activity, and SLS were defined for each frame based on the coordinates of the keypoints of body parts (i.e. head, thorax, abdomen, abdominal tip, left hind leg, and right hind leg) (Fig. 2). Locomotor activity was defined as the movement of a distance equal to or greater than body length within one frame. Feeding activity was defined when the head coordinates were located within the feeding area. SLS was defined as a change in the distance of the abdomen's coordinates within one frame (1 min) of less than three pixels and when this immobile state persisted for five or more frames (equivalent to five or more minutes). Criteria for >5 min of immobility were adopted from the definition of SLS in fruit flies (Shaw et al., 2000; Hendricks et al., 2000). Food and water were replenished and replaced three times during the 14 days of recording. As the position of the acrylic case shifts before and after these replenishments, the coordinates of the feed area were adjusted accordingly.

Raw data were visualised as double-plotted actograms using ActogramJ (Schmid et al., 2011). Periodicity analysis was conducted using the Chi-square periodogram (Sokolove and Bushell, 1978) and the LombScargle periodogram (Ruf, 1999). These approaches were selected to consider their characteristics and the risks of methods (Tackenberg and Hughey, 2021). If a peak in the periodogram exceeded a confidence level of 0.05 (α=0.05), it was considered as a significant rhythm (Kaneko et al., 2000). Oriana software, v.4 (Kovach Computing Wales, UK) was used to calculate the average acrophase (peak frequency of a certain behaviour within 24 h) during the experimental period (Kovach, 2011). To compare the levels of behaviours or posture (i.e. locomotor activity, SLS, and hind leg angle) during the light and dark periods, or for the analysis of hourly trends at each level, we calculated average number of frames determined with the behaviours or posture per 1 h or 12 h. We defined it as follows: (1) the number of frame-pairs between which a motion was detected during a 1 h or 12 h period was calculated, (2) the average of them over the same time slot over 2 weeks was calculated, and (3) this average value was defined as the average number of frames of locomotor activity per 1 h or 12 h period. For feeding activity, the averages for every 1 h and every 12 h were used because the total activity was small.

### Quantification of the angle between the abdomen and both hind legs

The coordinates of the abdomen, left hind legs, and right hind legs were used to calculate the angles hind leg opening. A Q-Q plot was used to check whether the angle datasets followed a normal distribution. The top and bottom 2.5% of the angle data were considered outliers and excluded, with the data within the 95% confidence interval used for further analysis. Postures in which the angles of leg opening were outliers included those in which the crickets brought their hind legs up to their mouths. To statistically assess the difference in the angle during the dark and light phases, the angles were averaged every hour, and the change over a day was calculated every hour and every 12 h. To investigate the relationship between the angles and the SLS, the linear regression correlation coefficient was computed between the angle and the SLS for each hour.

### Statistics

Student's *t*-test was used to compare differences in the means of activities between night and day. Two-way analysis of variance (ANOVA) followed by Sidak's multiple comparison test were used to test for differences in behavioural transition probabilities. The Shapiro-Wilk test and the Kolmogorov–Smirnov tests were used to determine whether the angle data followed a normal distribution. Linear regression analysis was used to demonstrate the correlation between the SLS and the angle between three points: the abdomen and both hind legs. Periodic and circular analyses were performed as described previously. Statistical significance was set at *P*<0.05.

## Supplementary Material

10.1242/biolopen.060237_sup1 Supplementary information
