# Supplementary material for: DeepLabCut-based daily behavioural and posture analysis in a cricket
Source: Biol Open. 2024 Apr 23;13(4):bio060237. doi: 10.1242/bio.060237 (PMC11070783; doi:10.1242/bio.060237)
Supplement: Supplementary information [file biolopen-13-060237-s1.pdf]

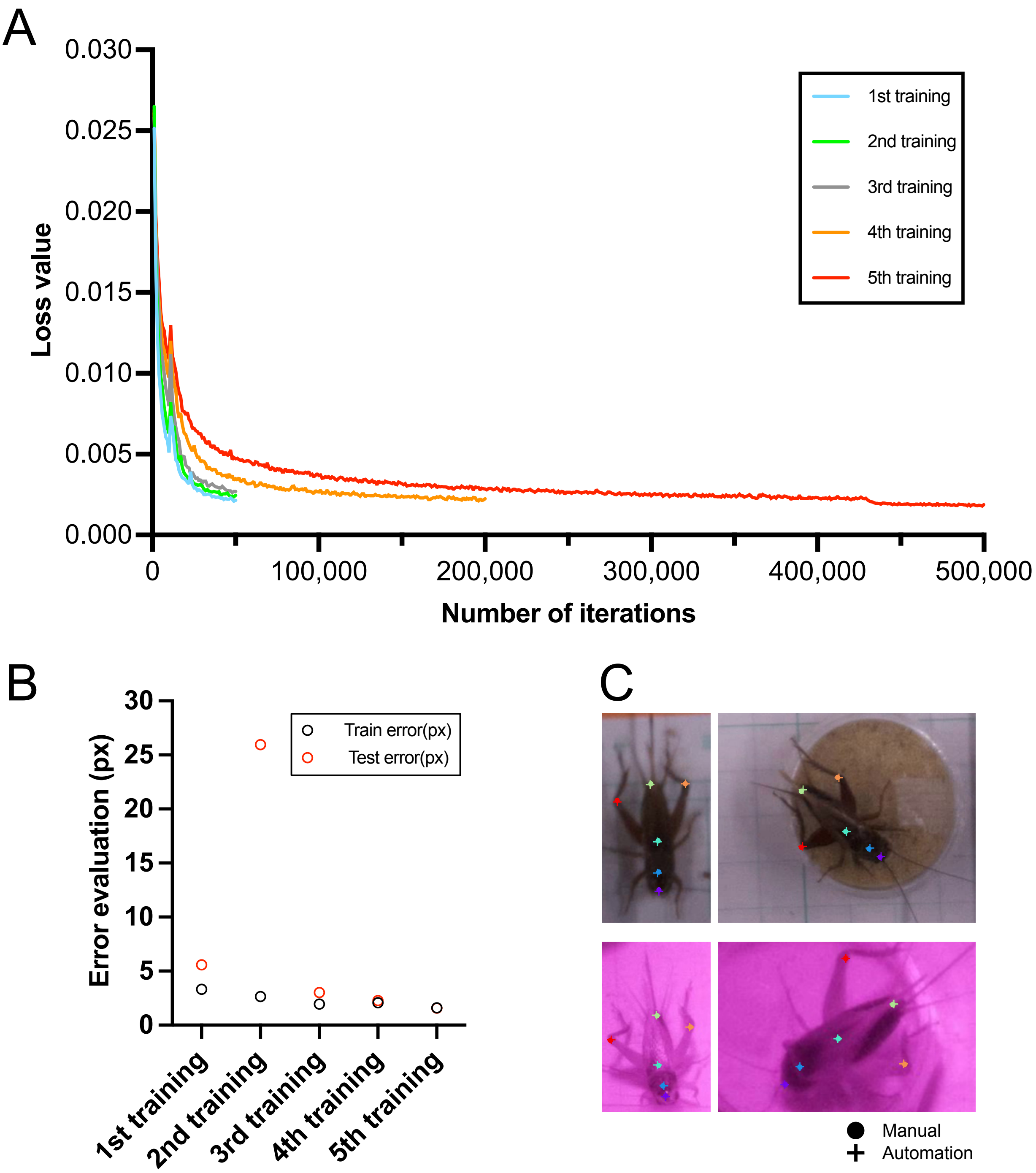
A
0.030
0.025
0.020
0.015
0.010
0.005
0.000
Loss value
0
100,000
200,000
300,000
400,000
500,000
Number of iterations
1st training
2nd training
3rd training
4th training
5th training
B
30
25
20
15
10
5
0
Error evaluation (px)
Train error(px)
Test error(px)
1st training
2nd training
3rd training
4th training
5th training
C
Manual
Automation

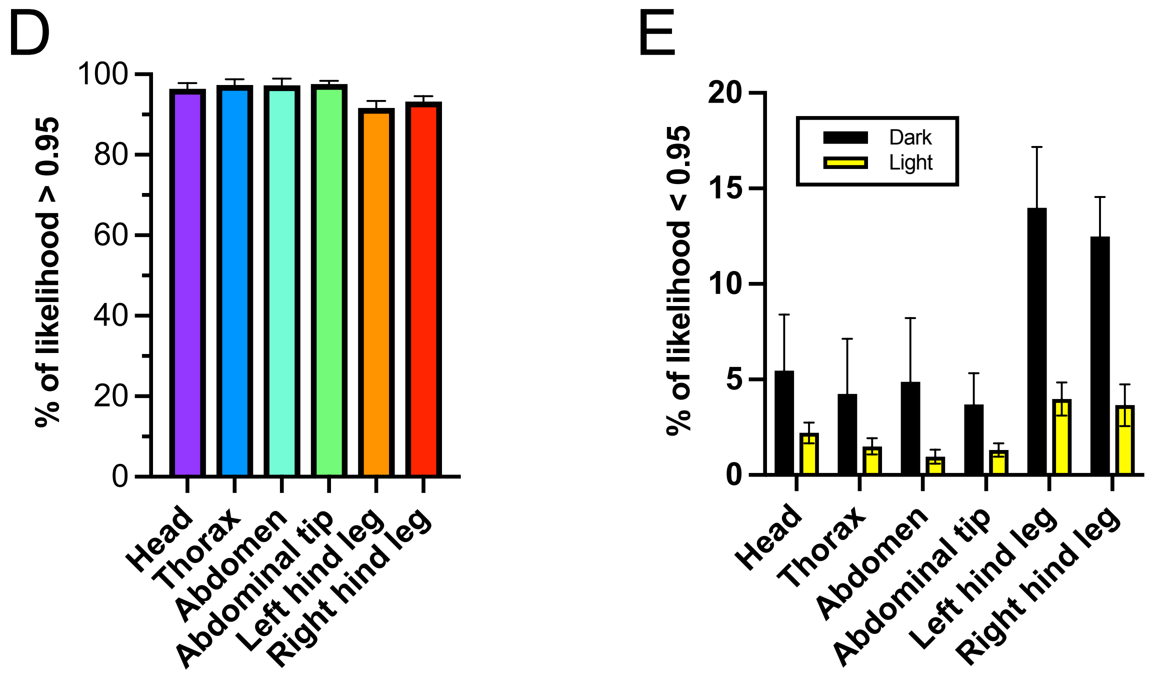

Fig. S1. Model accuracy evaluation. (A) The loss value was calculated based on the discrepancies between the model's predicted keypoint coordinates and the actual labelling data. This graph shows that the loss value decreases as the number of iterations increases. (B) The train error is calculated as the difference in distance (px) between the actual labelled coordinates and the model's predicted keypoint coordinates, averaged over 6 keypoints and the number of teacher data (black circles). Test error was calculated in the same way as Train error, using a dataset reserved for validating the model's performance (red circles). These graphs show the accuracy improved with an increased number of training iterations. (C) Example images of the train error by the model after the 5th training (manual: circles, automatic: crosses). (D) The percentage of labelling accuracies was calculated as the percentage of frames for which the likelihood of validity that was greater than or equal to 0.95 for each coordinate of the output raw data (N=6). (E) The missing value was calculated as the percentage of frames for which the likelihood was less than 0.95 and was indicated by light and dark phases for each keypoint (N=6). The error bars indicate standard error of the mean (SEM).

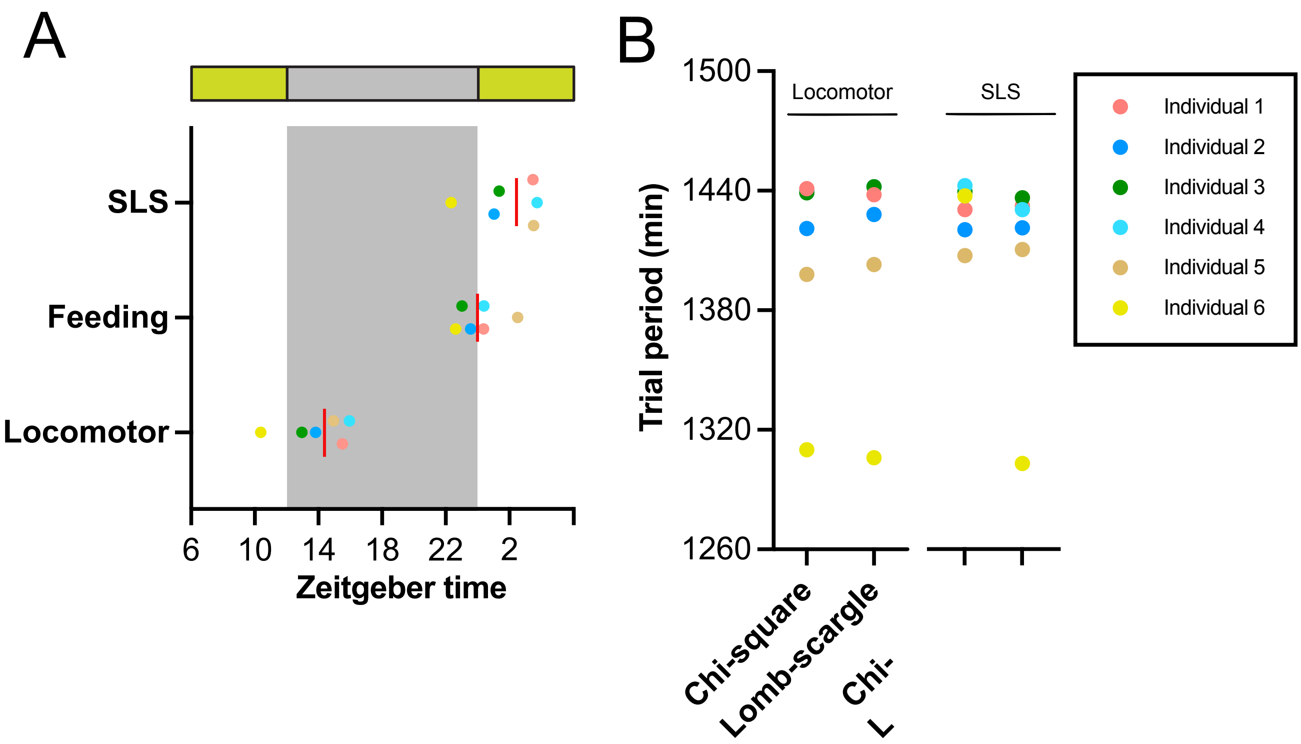

Fig. S2. Behavioural profile of each of the six individuals (Colours correspond to individual numbers). (A) Acrophase for locomotion, feeding activity, and SLS were calculated by angle mean and vector length weighting from circular analysis, respectively. The red line shows the median of each behaviour. (B) The periodograms for locomotion and SLS were created by Chi-square and Lomb-Scargle statistics. This graph demonstrated that the frequencies of both locomotion and SLS have a daily rhythm of approximately 24 h (1440 min), whereas individual #6 had a cycle shorter than 24 h.

Biology Open • Supplementary information

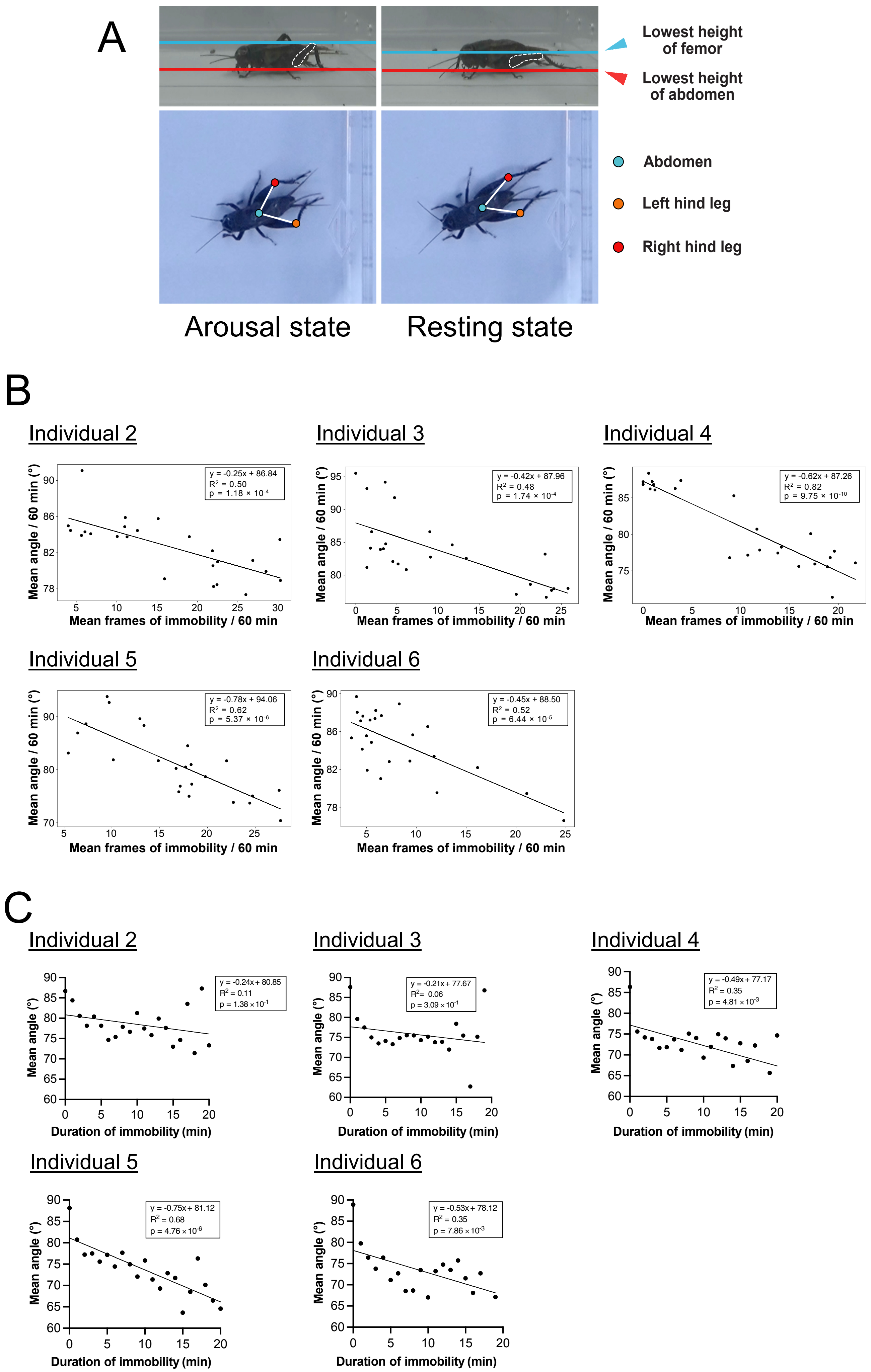

Biology Open • Supplementary information

Fig. S3. The body posture associated with the SLS in crickets. (A) Postural features at awake state (the left panels) and the SLS (the right panels) were captured from the side (the upper panels) and from above (the lower panels). The decrease in the height of femur and that of the abdomen, leg relaxation (calculated based on the angle between the three points of abdomen, left hind leg, and right hind leg) suggested that these postures are characteristic of the SLS. (B) A scatter plot of the mean angle per h against the mean SLS frequency corresponding to same hour was calculated with a fitted linear regression line in each individual. The equation of the line and Pearson's r value are provided, indicating a relatively negative correlation, with variation among individuals. (C) The mean angle was calculated for each number of continuous immobility frames from 0 to 20 with a fitted linear regression line (1 frame per minute). The equation of the line, with R squared as the coefficient of determination, is provided, indicating a relatively negative correlation in each individual.

Biology Open • Supplementary information

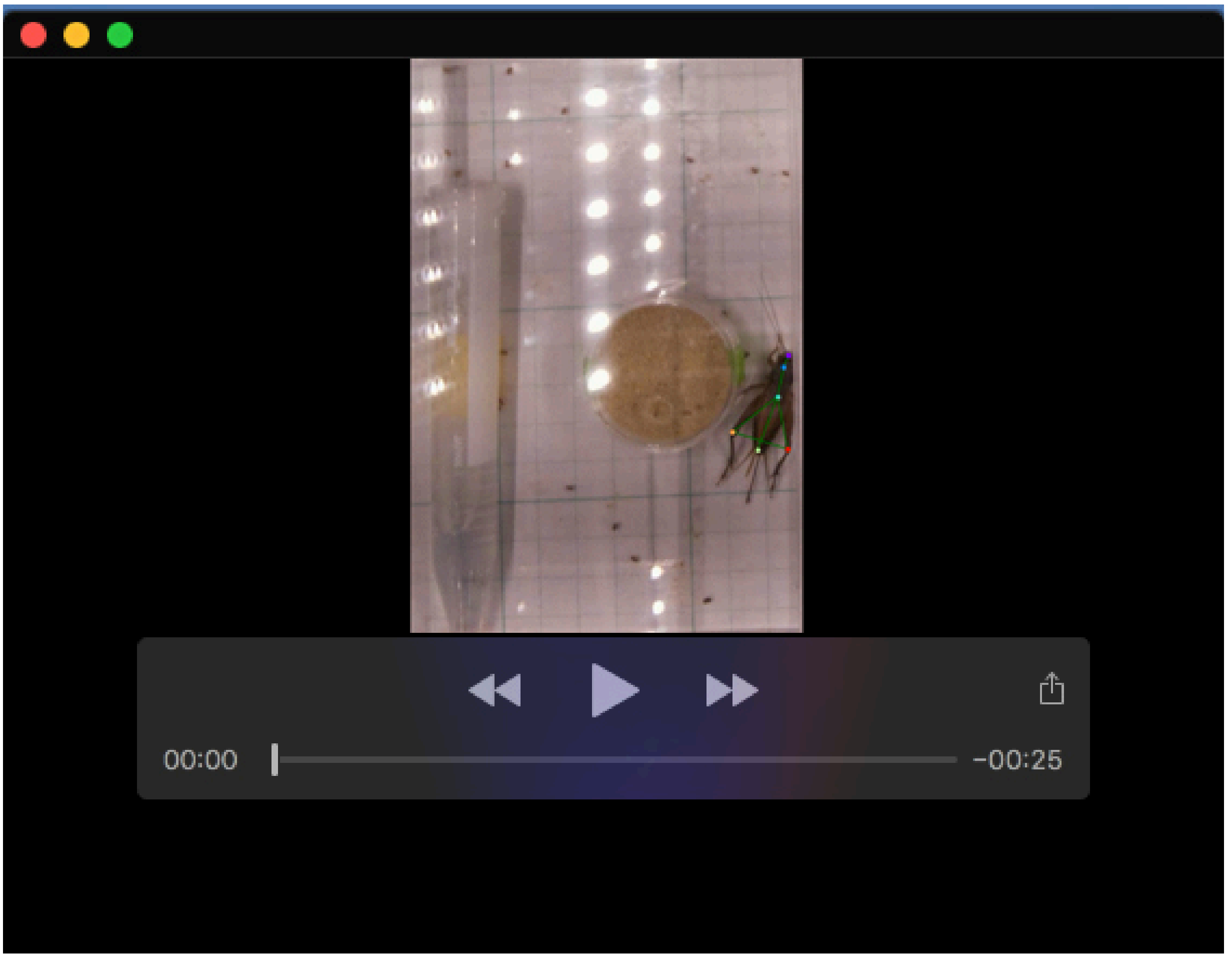

Movie 1. Labelled video output from DeepLabCut. Automatic labelling by the model after the fifth training showed high accuracy for the six keypoints of the crickets in both the dark and light phases, independent of the LEDs and objects.
